# Clinical Activity and Safety of Penpulimab (Anti-PD-1) With Anlotinib as First-Line Therapy for Unresectable Hepatocellular Carcinoma: An Open-Label, Multicenter, Phase Ib/II Trial (AK105-203)

**DOI:** 10.3389/fonc.2021.684867

**Published:** 2021-07-13

**Authors:** Chun Han, Sisi Ye, Chunhong Hu, Liangfang Shen, Qun Qin, Yuxian Bai, Shizhong Yang, Chunmei Bai, Aimin Zang, Shunchang Jiao, Li Bai

**Affiliations:** ^1^ Department of Medical Oncology, Chinese People's Liberation Army (PLA) General Hospital, Beijing, China; ^2^ Department of Oncology, The Second Xiangya Hospital, Central South University, Changsha, China; ^3^ Department of Oncology, Xiangya Hospital, Central South University, Changsha, China; ^4^ Department of Gastrointestinal Oncology, Harbin Medical University Cancer Hospital, Harbin, China; ^5^ Hepatopancreatobiliary Center, Beijing Tsinghua Changgung Hospital, Tsinghua University, Beijing, China; ^6^ Department of Oncology, Peking Union Medical College Hospital, Beijing, China; ^7^ Hebei Key Laboratory of Cancer Radiotherapy and Chemotherapy, Department of Medical Oncology, Affiliated Hospital of Hebei University, Baoding, China

**Keywords:** hepatocellular carcinoma, first-line treatment, immune checkpoint inhibitors, antiangiogenics, anlotinib, penpulimab

## Abstract

**Objective:**

This study aims to assess the efficacy and safety of penpulimab (a humanized anti-PD-1 IgG1 antibody) with anlotinib in the first-line treatment of Chinese patients with uHCC.

**Methods:**

In this open-label multicenter phase Ib/II trial, patients with histologically or cytologically confirmed uHCC, without previous systemic treatment, aged 18–75 years old, classified as BCLC stage B (not amenable for locoregional therapy) or C, with Child–Pugh score ≤7 and ECOG performance status ≤1 were enrolled. Patients received penpulimab [200 mg intravenous (i.v.) Q3W] and oral anlotinib (8 mg/day, 2 weeks on/1 week off). The primary endpoint was objective response rate (ORR). Secondary endpoints included safety, disease control rate (DCR), progression-free survival (PFS), time to progression (TTP), duration of response (DoR), and overall survival (OS). This trial is registered with ClinicalTrials.gov (NCT04172571).

**Results:**

At the data cutoff (December 30, 2020), 31 eligible patients had been enrolled and treated with a median follow-up of 14.7 months (range, 1.4–22.1). The ORR was 31.0% (95% CI, 15.3–50.8%), and the DCR was 82.8% (95% CI, 64.2–94.2%). The median PFS and TTP for 31 patients were 8.8 months (95% CI, 4.0–12.3) and 8.8 months (95% CI, 4.0–12.9) respectively. The median OS was not reached; the 12-month OS rate was 69.0% (95% CI, 48.9–82.5%). Only 19.4% (6/31) of patients had grade 3/4 treatment-related adverse events (TRAEs).

**Conclusion:**

Penpulimab plus anlotinib showed promising anti-tumor activity and a favorable safety profile as first-line treatment of patients with uHCC.

## Introduction

Primary liver cancer is the fourth leading cause of death from cancer worldwide with a 5-year survival rate of only 12–20% (1–3). Hepatocellular carcinoma (HCC) is the major histological subtype, accounting for approximately 80% of liver cancer. HBV infection is one of the main risk factor of HCC, and in China the HBV carrier rate was 7.18% varying from the low rate of 0.1–2.0% in the United States and Western Europe. Consequently, in excess of 50% of newly diagnosed cases and deaths from HCC have occurred in China (2, 3), approximately 70–80% of whom were related to HBV infection, quite distinct from Western patients (4, 5). Patients with HCC are often diagnosed at advanced stages, not amenable to surgery or locoregional treatment. Sorafenib and lenvatinib, previously demonstrated to prolong survival by a few months in patients with HCC (6–8), were the only two drugs approved by the Food and Drug Administration (FDA) until 2018 for first-line treatment of HCC.

Recently, considerable progress has been made in the treatment of HCC, especially with regard to immunotherapy. PD-1 (programmed cell death-1) inhibitors such as nivolumab and pembrolizumab have been shown to have promising anti-tumor activity in the second-line treatment of HCC and have been approved by the FDA for HCC patients, who were previously treated with sorafenib (9, 10). Nivolumab was compared to sorafenib in a first phase III clinical trial (CheckMate-459) of first-line immunotherapy for HCC (11). Although the study did not achieve its primary endpoint of over survival (OS), nivolumab showed clinical improvements on ORR (nivolumab *vs.* sorafinib: 15 *vs.* 7%).

Previous studies have shown that dual PD-1 and VEGF blockade may exert a synergistically inhibitory effect on tumor growth (12–14). The combination of immunotherapy and anti-angiogenic therapy has been proven to be an even better option than PD-1/L1 monotherapy and has become the preferred standard treatment option for HCC first-line therapy. Patients treated with atezolizumab combined with bevacizumab showed significantly better OS (19.2 *vs.* 13.4 months; HR, 0.66; 95% CI, 0.52–0.85) and progression free survival (PFS) (6.9 *vs.* 4.3 months; HR, 0.65; 95% CI, 0.53–0.81) than that of sorafenib in a global phase III randomized trial, IMbrave150 (15). As a result, atezolizumab plus bevacizumab became the first FDA-approved first-line immunotherapy-based treatment for HCC patients. Several first-line treatment trials that are still ongoing in recent years, such as the Study 117, a phase Ib study of lenvatinib plus nivolumab in patients with unresectable HCC, and Keynote 524, a phase 1b trial of lenvatinib plus pembrolizumab in unresectable HCC, both of which achieved an ORR over 30%, treated with lenvatinib plus nivolumab or pembrolizumab (16, 17). Moreover, pooled analysis showed that HBV-positive HCC patients have less clinical benefit compared to HBV-negative patients treated with immunotherapy, especially with PD-1/L1 monotherapy. In contrast, HBV-positive patients who received a combination of anti-PD-1/PD-L1 plus anti-VEGF therapy achieved similar ORRs and DCRs to those of HBV-negative patients (18), a combination therapy maybe more suitable for Chinese HCC patients.

Subgroup analysis of IMbrave150 highlighted the efficacy and safety of treatment with atezolizumab plus bevacizumab in the Chinese HCC population (19), and more trials of PD-1/L1 inhibitors combined with anti-angiogenic drugs for HCC in China are underway, but the sample size of which is small (20–22). Current research of first-line immunotherapy for HCC patients in China is relatively limited; there remains an urgent need for additional effective and safe treatments of HCC.

Penpulimab is a newly developed humanized high-affinity IgG1 anti-PD-1 monoclonal antibody by Akeso Biopharma intended for treatment of various malignancies. Penpulimab has a fragment crystallizable (Fc) mutation to eliminate Fc receptor and complement mediated effectors, which may attenuate effector activity such as ADCC (antibody-dependent cell-mediated cytotoxicity) or CDC (complement-dependent cytotoxicity) (23). Anlotinib, an oral tyrosine kinase inhibitor (TKI) that targets VEGFR 1 to 3, FGFR 1 to 4, PDGFR *α* and *β*, and c-Kit, has well-established efficacy and safety profiles in the treatment of numerous malignancies (24, 25).

The present study (AK-105-203) is a multicenter, phase 1/2 study aimed to assess the safety and efficacy of penpulimab combined with anlotinib in the first-line treatment of patients with unresectable HCC.

## Material and Methods

### Study Design and Patients

AK105-203 is a multicenter, open-label, phase Ib/II trial of penpulimab combined with anlotinib in patients with unresectable HCC without previous therapy. This study is registered with ClinicalTrials.gov (number NCT04172571).

Patients eligible for the trial were aged 18 to 75 years old, had a histologically or cytologically confirmed diagnosis of HCC, and a Barcelona Clinical Liver Cancer (BCLC) stage B or C, and were unsuitable to receive surgical or other local treatment. Eligible patients also had at least one measurable lesion as defined by the Response Evaluation Criteria in Solid Tumors (RECIST) version 1.1, an Eastern Cooperative Oncology Group (ECOG) performance status of 0 or 1, a predicted life expectancy greater than 3 months, adequate organ function, and a Child–Pugh score of 7 or less (Child–Pugh A or B7). Patients with chronic infections of hepatitis C virus (HCV) or hepatitis B virus (HBV) (viral load <500 IU/ml before enrollment) were also allowed to enroll.

Key exclusion criteria included previous systemic anti-tumor therapy, including cytotoxic therapy, targeted therapy, or immunotherapy. This study also excluded patients with fibrolamellar and mixed hepatocellular or cholangiocarcinoma subtypes of hepatocellular carcinoma; tumor invasion of the main portal vein, the inferior vena cava or cardiac involvement (determined by imaging); other active malignancies; esophageal or gastric variceal bleeding within the past 6 months; or a history of bleeding events.

### Procedures

Eligible patients received penpulimab and anlotinib until disease progression (according to RECIST version 1.1), unacceptable toxicity, patient withdrawal of consent, or investigator decision. Penpulimab was administered intravenously at a dose of 200 mg on day 1 of each 3-week cycle, for a maximum of 35 cycles (approximately 2 years). Anlotinib was given orally at a dose of 8 mg on day 1 through 14 of a 21-day cycle for a maximum of 35 cycles (approximately 2 years).

Patients could continue penpulimab monotherapy after disease progression if they met the following criteria, patients may have possible clinical benefit as assessed by the investigator; could tolerate the toxicity of the drug; have stable ECOG performance status; do not have serious complications such as central nervous system metastases requiring urgent alternative medical interventions. Patients were well informed prior to continuing treatment.

The initial tumor imaging was performed within 21 days prior to the first intervention dose. Thereafter, the response was assessed every 6 weeks according to RECIST version 1.1 by the investigators. If patients discontinued treatment due to a cause other than disease progression or death, tumor response evaluation would continue until new anti-tumor therapy is initiated, disease progression, death, loss of follow-up, or withdrawal of informed consent. Patients were scanned for OS every 12 weeks until death, withdrawal of consent from participation in the study, or the end of the study (whichever occurred first).

### Safety Assessment

Data on adverse events (AEs) and laboratory abnormalities were collected from the time of treatment allocation until 30 days following treatment cessation (90 days for serious adverse events). AEs were graded using the National Cancer Institute Common Terminology Criteria for Adverse Events, version 4.0. Immune-related adverse events, defined as adverse events associated with penpulimab exposure that were consistent with immune phenomena and that had a potentially immunological cause, were prespecified as events of interest.

### Outcomes

The primary endpoint was the objective response rate (ORR), defined as the proportion of participants who achieved partial response or complete response. Secondary endpoints were safety, disease control rate (DCR), progress-free survival (PFS), time to progression (TTP), duration of response (DoR) and overall survival (OS). DCR was defined as the proportion of participants with complete and partial responses plus stable disease with a duration of at least 6 weeks. PFS was defined as the time from treatment allocation to the first documented disease progression or death from any cause, whichever occurred first. TTP was defined as the time from first day of treatment to the date of first documented disease progression. DoR was defined as the time from first confirmed complete or partial response to disease progression or death. OS was defined as the time from the first dose of study medication to death from any cause. ORR and DCR were assessed in participants who underwent at least two efficacy evaluations after the first dose of study medication. DoR was evaluated in responders. PFS, TTP, OS, and safety profiles were assessed in all participants who received at least one dose of penpulimab in combination with anlotinib.

### Statistical Analysis

Recent studies have shown that the median ORR of first-line treatment with sorafenib was less than 10%. An estimated ORR of at least 30% treated by penpulimab with anlotinib was considered to be clinically meaningful. Using *α* = 0.05, a power of 80%, a loss to follow-up of 20%, and based on an estimated ORR of 30%, the minimum sample size was estimated to be 30.

We summarized patient characteristics, safety analysis, and anti-tumor activity descriptively by using medians (95% CI) for quantitative variables and numbers (percentages) for qualitative variables. The 95% CI based on binomial distribution was constructed for the calculated ORR and DCR. We used the Kaplan–Meier method to determine medians and 95% CIs for TTP, PFS, DoR, and OS. All statistical analyses were performed using the SAS version 9.4 software (SAS Institute, Cary, NC, USA). The data cutoff date was December 30, 2020.

## Results

**Figure 1:**
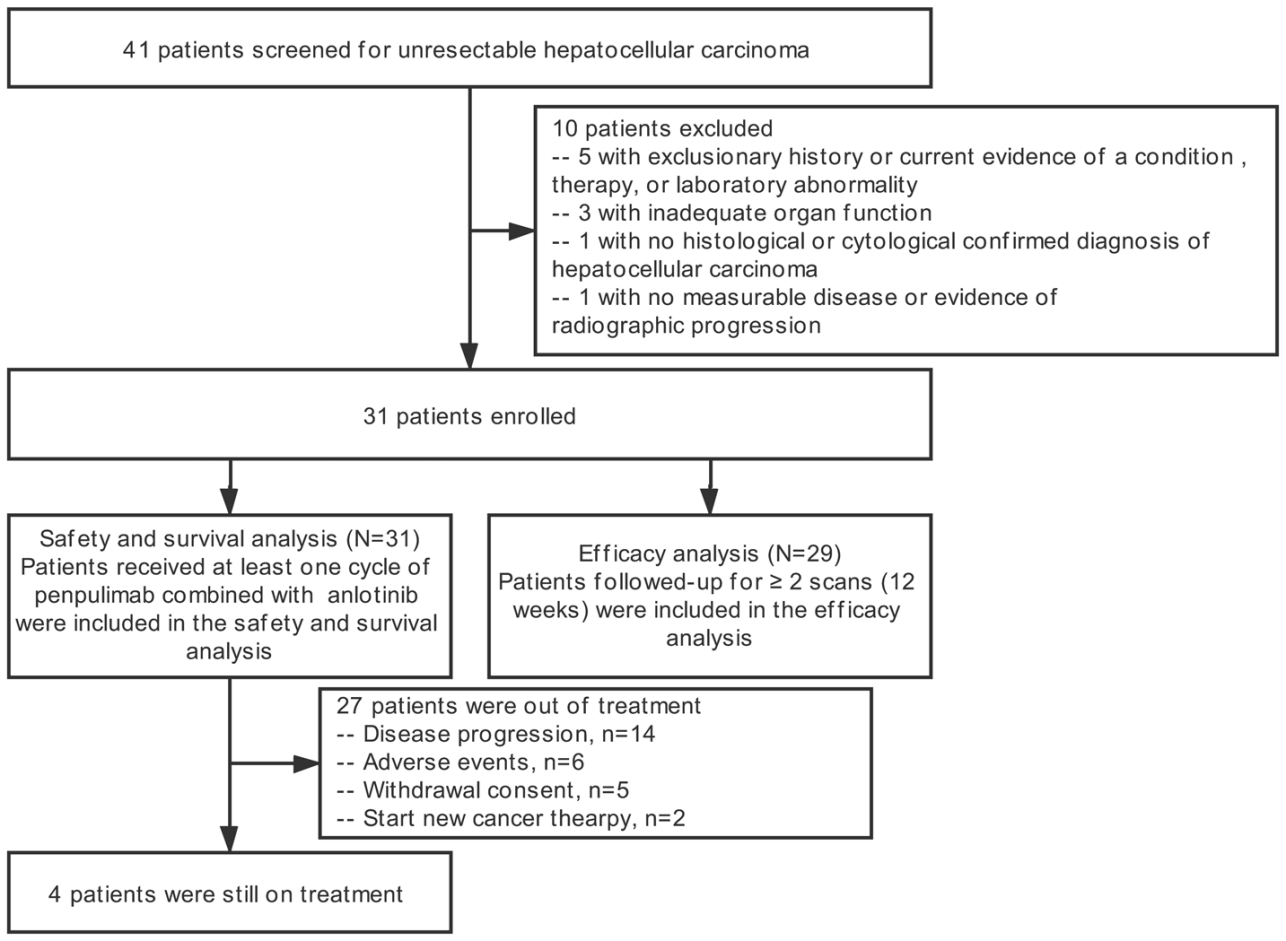
Trial profile.

Between January 24, 2019 and December 13, 2019, 41 patients screened at eight centers in China for eligibility, of whom10 patients failed to meet inclusion criteria. At the data cutoff (December 31, 2020), of the 31 patients who received at least one dose of the assigned treatment, 27 patients were out of treatment (14 patients ended the treatment because of tumor progression, 6 patients because of TEAEs (Treatment-Emergent Adverse Events), 5 patients withdrew informed consent, 2 patients because of new anti-tumor therapy (**Figure 1**).

**Figure 2:**
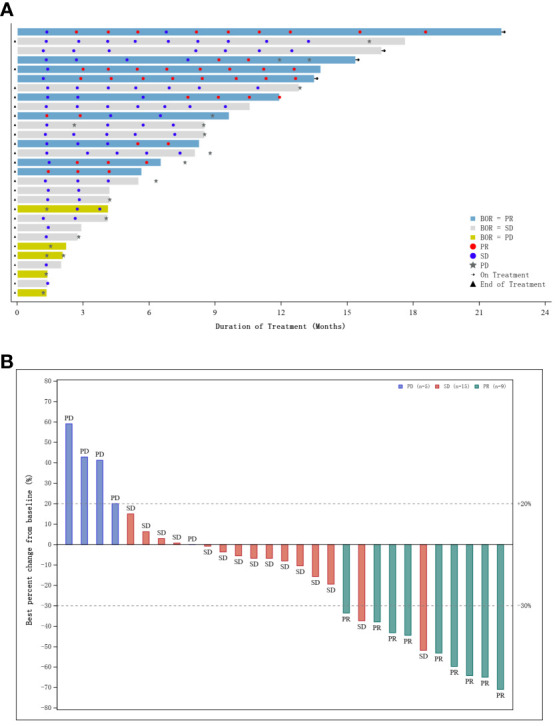
**(A)** Swimmer plot of patients who underwent at least two post-baseline tumor assessments; **(B)** Waterfall plot of best percentage change from baseline in sum of diameters with best overall response.

**Table 1** shows the baseline characteristics of the 31 enrolled participants. At analysis, 29 patients were included in the efficacy analysis cohort (**Table 2**). At database cutoff, the median follow-up was 14.7 months (range, 1.4–22.1) for all enrolled patients and 15.2 months (range, 3.7–22.1) for efficacy analysis cohort respectively. The ORR was 31.0% (95% CI, 15.3–50.8%) based on RECIST version 1.1 guidelines. Of the responders, six patients achieved a PR in 3 months (**Figure 2A**), DoRs ranged from 2.8+ to 15.9+ months. One responder withdrew the trial due to obstructive jaundice. In terms of the best overall response, nine patients (31.0%) had a partial response, fifteen (51.7%) had stable disease, and five (17.2%) had tumor progression, yielding a DCR of 82.8% (95% CI, 64.2–94.2%). The tumor size was reduced with treatment in more than two-thirds of patients (**Figure 2B**).

**Table 1:** Baseline clinical characteristics.

Characteristic	All patients (N = 31)
**Median age (range)**	56 (23–74)
**Sex—No. (%)**	
Male	25 (80.6)
Female	6 (19.4)
**ECOG PS—No. (%)**	
0	20 (64.5)
1	11 (35.5)
**Child–Pugh—No. (%)**	
A	31 (100.0)
B	0 (0.0)
**BCLC—No. (%)**	
B	7 (22.6)
C	24 (77.4)
**Infection—No. (%)**	
Hepatitis B virus	19 (61.3)
Hepatitis C virus	2 (6.5)
Non-infected	9 (29.0)
Unknown	1 (3.2)
**AFP, ng/ml—No. (%)**	
>400	9 (29.0)
≤400	22 (71.0)
**Extrahepatic spread—No. (%)**	
Yes	19 (61.3)
No	12 (38.7)

**Table 2:** Responses to study medication (n = 29).

Response parameters n (%)	Anlotinib + Penpulimab (N = 29)
Median follow-up (range)**—**months	15.2 (3.7–22.1)
**Best overall response**	
Complete Response (CR)	0
Partial Response (PR)	9 (31.0%)
Stable Disease (SD)	15 (51.8%)
Progressive Disease (PD)	5 (17.2%)
**ORR, % (95% CI)**	31.0% (15.3–50.8%)
**DCR, % (95% CI)**	82.8% (64.2–94.2%)

**Figure 3:**
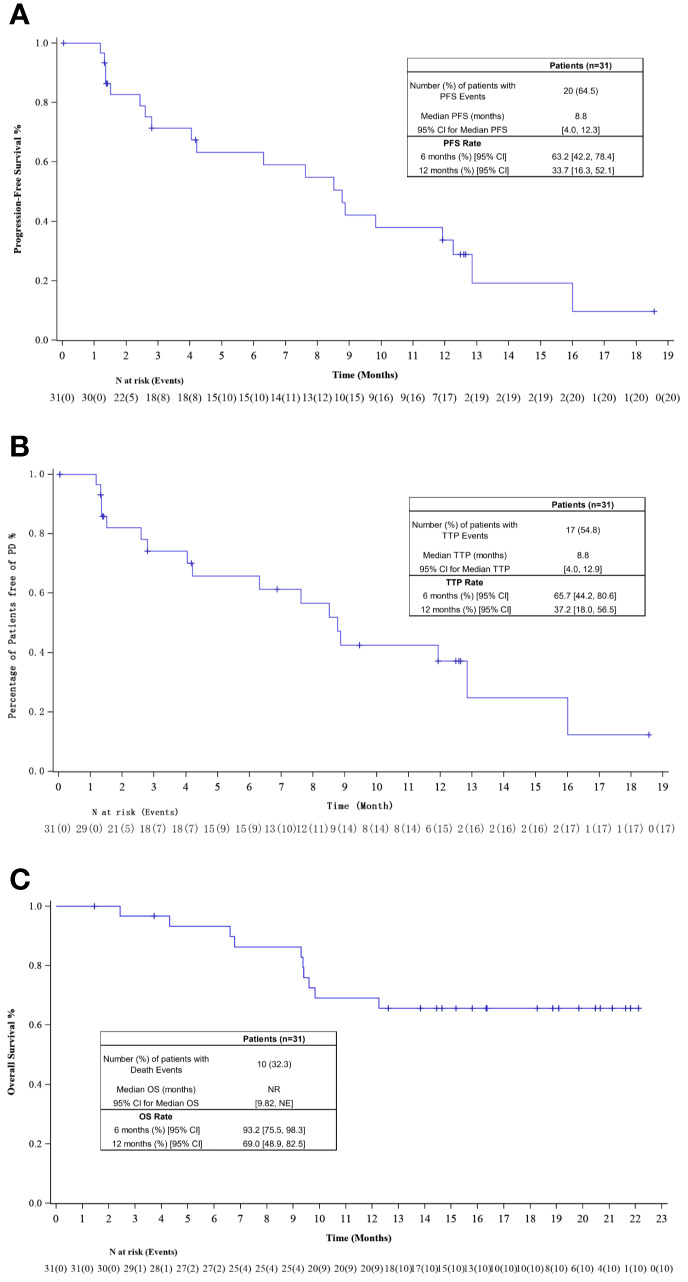
**(A)** Kaplan–Meier plot of progression-free survival (PFS); **(B)** Kaplan–Meier plot of time to progression (TTP); **(C)** Kaplan–Meier plot of overall survival (OS).

The investigator-assessed median PFS was 8.8 months (95% CI, 4.0–12.3), with an estimated 12-month PFS rate of 33.7% (95% CI, 16.3–52.1%; **Figure 3A**). Median TTP for 31 patients was 8.8 months (95% CI, 4.0–12.9), with an estimated rate at 12 months of 37.2% (95% CI, 18.0–56.5%; **Figure 3B**). The median OS had not been reached yet, the estimated 6- and 12-month OS rate was 93.2% (95% CI, 75.5–98.3%) and 69.0% (95% CI, 48.9–82.5%; **Figure 3C**).

At least one TEAE (treatment emergent adverse event) was reported in 96.8% (30/31) of participants; serious AEs were reported in 19.4% (6/31) of patients (**Table 3**). TEAEs led to death in three (9.7%) patients and to discontinuation of treatment in seven (22.6%) patients. No treatment-related deaths were reported in the study. Treatment-related AEs (TRAE) were reported in 90.3% (28/31) of patients, the most common of which occurred in at least 10% of the participants were elevations in levels of aspartate aminotransferase [12 (38.7%)], alanine aminotransferase [11 (35.5%)], conjugated bilirubin [7 (22.6%)], blood bilirubin [7 22.6%)], decreased platelet counts [7 (22.6%)], asthenia [7 (22.6%)], and rash [5 (16.1%)] (**Table 4**). TRAEs of grade 3 or above were reported in 19.4% (6/31) patients including hypertension [2 (6.5%)], rash [1 (3.2%)], rash generalized [1 (3.2%)], chest pain [1 (3.2%)], peripheral swelling [1 (3.2%)] and hepatic rupture [1 (3.2%)] (**Table S1**). Immune-related AEs of grade 3 or higher occurred in 6.5% (2/31) of patients, rash in two patients. Additional TEAE information is included in **Table S1**.

**Table 3:** Overview of adverse events (AEs).

	Patients (N = 31), n (%)
TEAEs	30 (96.8)
≥Grade 3 TEAEs	13 (41.9)
TRAEs	28 (90.3)
≥Grade 3 TRAEs	6 (19.4)
irAE	13 (41.9)
≥Grade 3 irAEs	2 (6.5)
Serious AE	6 (19.4)
Serious TRAEs	2 (6.5)
TEAE Leading to any study drug discontinuation	7 (22.6)
TEAE Leading to death	3 (9.7)
TRAEs leading to study drug discontinuation	4 (12.9)
TRAEs leading to death	0

TEAE, treatment emergent adverse event; TRAE, treatment related adverse event; irAE, Immune-related adverse event.

**Table 4:** Treatment related AEs (TRAEs) with an incidence of more than 10%.

	Patients (N = 31), n (%)
**Patients with at least oneTRAE**	28 (90.3)
Aspartate aminotransferase increased	12 (38.7)
Alanine aminotransferase increased	11 (35.5)
Bilirubin conjugated increased	7 (22.6)
Blood bilirubin increased	7 (22.6)
Platelet count decreased	7 (22.6)
Asthenia	7 (22.6)
Rash	5 (16.1)
Blood pressure increased	4 (12.9)
Dysphonia	4 (12.9)
Hypothyroidism	4 (12.9)

## Discussion

Penpulimab combined with anlotinib for the treatment of unresectable Chinese HCC demonstrated the statistically defined clinical benefit with an ORR of 31% and DCR of 82.8%. To put into context with the previous clinical trial IMbrave150, atezolizumab plus bevacizumab for HCC as first-line treatment of Chinese HCC achieved an ORR of 25% (19). This study achieved a similar efficacy compared to that of IMbrave150. Moreover, the overall ORRs of other first-line combination therapies in trials that are still ongoing have ranged from 29 to 54.2%, and those studies have been based primarily on western HCC patients (15–17, 19, 20, 26). In the current study, more than 60% of the patients were HBV-positive, yet achieved an ORR of 31%, similar to the results of previous studies. The responses seemed durable with the median TTP and PFS >8.8 months, longer than that of atezolizumab plus bevacizumab or camrelizumab plus apatinib (15, 27).

Penpulimab combined with anlotinib seemed to show a satisfactory safety profile, similar to other immunotherapy, with no unexpected AEs related to either single agent (28, 29). Owing to adverse events, 19.4% of patients ended the treatment. Most patients had grade 1–2 TRAEs; TRAEs of grade 3 or higher were reported in only 19.4% of patients, a relative lower rate than that reported in IMbrave150 or other studies (15–17). TRAEs that occurred in more than 10% of participants were primarily liver-related toxicities reflected in enzyme or bilirubin elevations, consistent with AEs seen in other trials (15, 27). Few participants experienced immune-mediated adverse events.

Evidence has increasingly accumulated that anti-angiogenic therapy exerts immunomodulatory effects on the tumor microenvironment, which appears to enhance the efficacy of anti-PD-1 monoclonal antibody by normalizing abnormal tumor vessels and increasing the infiltration of immune effector cells into tumors (14, 30, 31). Previous studies have shown that anlotinib could increase infiltration of innate immune cells, conferring significant synergistic therapeutic benefit when combined with immunotherapy (32). In addition, Noman found that inhibiting angiogenesis induced hypoxia in tumor tissue, which in turn upregulated PD-L1 expression on macrophages, dendritic cells, and tumor cells (33). Therefore, the administration of anti-vascular therapy could reasonably enhance the efficacy of immunotherapy. In this study, we further confirmed that immunotherapy combined with anti-angiogenic therapy appears to have promising clinical benefit.

Our pilot study has several limitations. As is typical of early phase clinical trials, the sample of subjects was relatively small; only 60% of patients were HBV infected compared to 80% in the Chinese HCC population. The result needs further validation with larger sample size.

This study further confirmed that a combination of a PD-1 inhibitor with an anti-VEGFR TKI has favorable clinical efficacy and acceptable toxicity in the treatment of unresectable HCC in patients not previously treated with systemic therapy, and penpulimab combined with anlotinib may be an optional regime. More data from updated clinical trials are needed to confirm these observations, and long-term clinical outcomes are being evaluated.

## Data Availability Statement

The original contributions presented in the study are included in the article/**Supplementary Material**. Further inquiries can be directed to the corresponding authors.

## Ethics Statement

The studies involving human participants were reviewed and approved by Ethics committee of Chinese PLA General Hospital; Ethics committee of The Second Xiangya Hospital, Central South University; Ethics committee of Xiangya Hospital, Central South University; Ethics committee of Harbin Medical University Cancer Hospital; Ethics committee of Beijing Tsinghua Changgung Hospital; Ethics committee of Peking Union Medical College Hospital; Ethics committee of Affiliated Hospital of Hebei University; Ethics committee of Zhongshan Hospital, Fudan University. The patients/participants provided their written informed consent to participate in this study.

## Author Contributions

LB, SJ, and CHa were involved in the trial design, interpretation of the results, drafting of the manuscript, and critical revision of the manuscript. SYe conducted the data analyses and assisted in the interpretation of the results. CHu, LS, QQ, YB, SYa, CB, and AZ participated in the patients’ selection and data extraction. All authors contributed to the article and approved the submitted version.

## Funding

National Key Research and Development (R&D) Plan (2016YFC1303602).

## Conflict of Interest

The authors declare that the research was conducted in the absence of any commercial or financial relationships that could be construed as a potential conflict of interest.

## Supplementary Material

Supplementary Table 1 Treatment related adverse events (TRAEs) by system organ class.
