# Supplementary material for: Clinical Activity and Safety of Penpulimab (Anti-PD-1) With Anlotinib as First-Line Therapy for Unresectable Hepatocellular Carcinoma: An Open-Label, Multicenter, Phase Ib/II Trial (AK105-203)
Source: Front Oncol. 2021 Jul 13;11:684867. doi: 10.3389/fonc.2021.684867 (PMC8313824; doi:10.3389/fonc.2021.684867)
Supplement: Supplementary Table 1 — Treatment related adverse events (TRAEs) by system organ class. [file Table_1.docx]

**Table s1: Treatment related Adverse Events(TRAEs) by System Organ Class**

| **TRAEs** | **Any grade, n (%)** | **Grade 3-5, n (%)** |
| --- | --- | --- |
| Patients with at least one TRAE | 28 (90.3) | 6 (19.4) |
| **Investigations** | **24 (77.4)** |  |
| Aspartate aminotransferase increased | 12 (38.7) |  |
| Alanine aminotransferase increased | 11 (35.5) |  |
| Bilirubin conjugated increased | 7 (22.6) |  |
| Blood bilirubin increased | 7 (22.6) |  |
| Platelet count decreased | 7 (22.6) |  |
| Blood pressure increased | 4 (12.9) |  |
| Blood lactate dehydrogenase increased | 3 (9.7) |  |
| Blood thyroid stimulating hormone decreased | 3 (9.7) |  |
| Blood thyroid stimulating hormone increased | 3 (9.7) |  |
| Gamma-glutamyltransferase increased | 3 (9.7) |  |
| Total bile acids increased | 3 (9.7) |  |
| White blood cell count decreased | 3 (9.7) |  |
| C-reactive protein increased | 2 (6.5) |  |
| Electrocardiogram QT prolonged | 2 (6.5) |  |
| Lipids increased | 2 (6.5) |  |
| Protein urine present | 2 (6.5) |  |
| Blood alkaline phosphatase increased | 1 (3.2) |  |
| Blood calcium decreased | 1 (3.2) |  |
| Blood creatinine increased | 1 (3.2) |  |
| Blood phosphorus decreased | 1 (3.2) |  |
| Blood urea increased | 1 (3.2) |  |
| Blood urine present | 1 (3.2) |  |
| Electrocardiogram T wave abnormal | 1 (3.2) |  |
| Electrocardiogram T wave amplitude decreased | 1 (3.2) |  |
| Eosinophil count abnormal | 1 (3.2) |  |
| Eosinophil percentage abnormal | 1 (3.2) |  |
| Haemoglobin decreased | 1 (3.2) |  |
| Neutrophil count decreased | 1 (3.2) |  |
| Plateletcrit decreased | 1 (3.2) |  |
| Prothrombin time prolonged | 1 (3.2) |  |
| Thyroglobulin increased | 1 (3.2) |  |
| Thyroxine decreased | 1 (3.2) |  |
| Thyroxine free decreased | 1 (3.2) |  |
| Thyroxine free increased | 1 (3.2) |  |
| Thyroxine increased | 1 (3.2) |  |
| Tri-iodothyronine free decreased | 1 (3.2) |  |
| Tri-iodothyronine free increased | 1 (3.2) |  |
| Tri-iodothyronine uptake increased | 1 (3.2) |  |
| Weight decreased | 1 (3.2) |  |
| **Gastrointestinal disorders** | **11 (35.5)** |  |
| Abdominal distension | 3 (9.7) |  |
| Diarrhoea | 3 (9.7) |  |
| Gastrointestinal hypomotility | 3 (9.7) |  |
| Abdominal pain | 1 (3.2) |  |
| Abdominal pain lower | 1 (3.2) |  |
| Dyspepsia | 1 (3.2) |  |
| Nausea | 1 (3.2) |  |
| Stomatitis | 1 (3.2) |  |
| **Skin and subcutaneous tissue disorders** | **11 (35.5)** |  |
| Rash | 5 (16.1) | 1 (3.2) |
| Palmar-plantar erythrodysaesthesia syndrome | 3 (9.7) |  |
| Pruritus | 2 (6.5) |  |
| Alopecia | 1 (3.2) |  |
| Eczema | 1 (3.2) |  |
| Rash generalised | 1 (3.2) | 1 (3.2) |
| **General disorders and administration site conditions** | **10 (32.3)** |  |
| Asthenia | 7 (22.6) |  |
| Oedema peripheral | 2 (6.5) |  |
| Pyrexia | 2 (6.5) |  |
| Chest pain | 1 (3.2) | 1 (3.2) |
| Face oedema | 1 (3.2) |  |
| Peripheral swelling | 1 (3.2) | 1 (3.2) |
| **Respiratory, thoracic and mediastinal disorders** | **7 (22.6)** |  |
| Dysphonia | 4 (12.9) |  |
| Cough | 2 (6.5) |  |
| Laryngeal discomfort | 2 (6.5) |  |
| Pneumonitis | 1 (3.2) |  |
| **Endocrine disorders** | **5 (16.1)** |  |
| Hypothyroidism | 4 (12.9) |  |
| Hyperthyroidism | 2 (6.5) |  |
| **Nervous system disorders** | **4 (12.9)** |  |
| Headache | 2 (6.5) |  |
| Dizziness | 1 (3.2) |  |
| Somnolence | 1 (3.2) |  |
| **Cardiac disorders** | **3 (9.7)** |  |
| Atrial fibrillation | 1 (3.2) |  |
| Atrial flutter | 1 (3.2) |  |
| Sinus tachycardia | 1 (3.2) |  |
| Supraventricular extrasystoles | 1 (3.2) |  |
| **Vascular disorders** | **3 (9.7)** |  |
| Hypertension | 3 (9.7) | 2 (6.5) |
| **Metabolism and nutrition disorders** | **2 (6.5)** |  |
| Decreased appetite | 1 (3.2) |  |
| Hypoproteinaemia | 1 (3.2) |  |
| **Ear and labyrinth disorders** | **1 (3.2)** |  |
| Tinnitus | 1 (3.2) |  |
| **Eye disorders** | **1 (3.2)** |  |
| Vision blurred | 1 (3.2) |  |
| **Injury, poisoning and procedural complications** | **1 (3.2)** |  |
| Hepatic rupture | 1 (3.2) | 1 (3.2) |
| **Musculoskeletal and connective tissue disorders** | **1 (3.2)** |  |
| Arthralgia | 1 (3.2) |  |
| **Reproductive system and breast disorders** | **1 (3.2)** |  |
| Menstrual disorder | 1 (3.2) |  |
